# Supplementary material for: Predicting risk of suicidal behaviour after initiation of selective serotonin reuptake inhibitors in children, adolescents and young adults: protocol for development and validation of clinical prediction models
Source: BMJ Open. 2023 Aug 23;13(8):e072834. doi: 10.1136/bmjopen-2023-072834 (PMC10450049; doi:10.1136/bmjopen-2023-072834)
Supplement: Author's manuscript [file bmjopen-2023-072834.draft_revisions.pdf]

BMJ Open

BMJ Open is committed to open peer review. As part of this commitment we make the peer review history of every article we publish publicly available.

When an article is published we post the peer reviewers' comments and the authors' responses online. We also post the versions of the paper that were used during peer review. These are the versions that the peer review comments apply to.

The versions of the paper that follow are the versions that were submitted during the peer review process. They are not the versions of record or the final published versions. They should not be cited or distributed as the published version of this manuscript.

BMJ Open is an open access journal and the full, final, typeset and author-corrected version of record of the manuscript is available on our site with no access controls, subscription charges or pay-per-view fees (http://bmjopen.bmj.com).

If you have any questions on BMJ Open's open peer review process please email info.bmjopen@bmj.com

BMJ Open

Predicting risk of suicidal behaviour after initiation of selective serotonin reuptake inhibitors in children, adolescents, and young adults: protocol for development and validation of clinical prediction models

Journal:	*BMJ Open*
Manuscript ID	bmjopen-2023-072834
Article Type:	Protocol
Date Submitted by the Author:	15-Feb-2023
Complete List of Authors:	Lagerberg, Tyra ; University of Oxford, Department of Psychiatry; Karolinska Institute, Department of Medical Epidemiology and Biostatistics<br>Virtanen, Suvi; Karolinska Institutet, Department of Medical Epidemiology and Biostatistics<br>Kuja-Halkola, Ralf; Karolinska Institutet, Department of Medical Epidemiology and Biostatistics<br>Hellner, Clara; Karolinska Institute, Clinical neuroscience<br>Lichtenstein, Paul; Karolinska Institutet, Department of Medical Epidemiology and Biostatistics<br>Fazel, Seena; University of Oxford, Department of Psychiatry; Oxford Health NHS Foundation Trust<br>Chang, Zheng; Karolinska Institutet, Department of Medical Epidemiology and Biostatistics
Keywords:	Suicide & self-harm < PSYCHIATRY, Child & adolescent psychiatry < PSYCHIATRY, MENTAL HEALTH, REGISTRIES, Risk Factors

SCHOLARONE™
Manuscripts

Predicting risk of suicidal behaviour after initiation of selective serotonin reuptake inhibitors in children, adolescents, and young adults: protocol for development and validation of clinical prediction models

Tyra Lagerberg[1,2], Suvi Virtanen[1], Ralf Kuja-Halkola[1], Clara Hellner[3,4], Paul Lichtenstein[1], Seena Fazel[2,5], Zheng Chang[1]

[1] Department of Medical Epidemiology and Biostatistics, Karolinska Institutet, Stockholm, Sweden

[2] Department of Psychiatry, Warneford Hospital, University of Oxford, Oxford, United Kingdom

[3] Department of Clinical Neuroscience, Centre for Psychiatry Research, Karolinska Institutet

[4] Stockholm Health Care Services, Stockholm, Sweden

[5] Oxford Health NHS Foundation Trust

Corresponding author:

Tyra Lagerberg. Email: tyra.lagerberg@psych.ox.ac.uk; Address: Department of Psychiatry, Warneford Hospital, University of Oxford, Oxford, United Kingdom OX3 7JX; Telephone: + 44 (0)1865 618200; ORCID: 0000-0003-2480-0216

Key words: prediction, selective serotonin reuptake inhibitors, suicidal behaviour, children and adolescents, young adults

Word count: 2,129

For peer review only - http://bmjopen.bmj.com/site/about/guidelines.xhtml

ABSTRACT

Introduction: There is concern regarding suicidal behaviour risk during selective serotonin reuptake inhibitor (SSRI) treatment, particularly among the young. A clinically useful model for predicting the risk of suicidal behaviour following SSRI initiation should have high predictive performance in terms of discrimination and calibration; additionally, transparency and ease of implementation are desirable.

Methods and analysis: Using Swedish national registers, we will identify all individuals initiating an SSRI aged 8-24 years 2008-2020. We will develop two models: a model based on a broad set of predictors, and a model based on a restricted set of predictors. For the broad predictor model, we will consider an ensemble of four machine learning models: XGBoost (XG), neural net (NN), logistic regression (LR), and support vector machine (SVM). The predictors with the greatest contribution to predictive performance will be determined. Clinical and statistical input will be used to select predictors from those with the highest rank, and included in each of the XG, NN, LR, and SVM models. An ensemble prediction will additionally be performed. If there is evidence of superiority in predictive performance as defined by the area under the receiver-operator curve (AUC) of any of the individual prediction models or the ensemble prediction, the model with highest predictive performance will be selected as the final restricted predictor model; otherwise, the LR model will be selected. All models will be tested in an unseen subset of the derivation data, chosen based on calendar year.

The aims are to A) develop a prediction model on risk of suicidal behaviour after initiation of SSRIs among children and youths using machine learning methods, and B) develop a clinically applicable model with a restricted set of predictors where transparency and scalability are favored.

Ethics and dissemination: The research is approved by the Swedish Ethical Review Authority (2020-06540). We will disseminate findings by publishing in peer-reviewed open-access journals, and presenting at international conferences.

STRENGTHS AND LIMITATIONS OF THIS STUDY

- The study will make use of a large and nationally representative data linkage of Swedish registers, covering virtually any Swedish resident who initiated SSRI treatment during the study period
- We will compare different machine learning models and approaches to selecting predictors, in order to explore the performance of an ensemble prediction model using machine learning methods in Swedish register data, and to develop a clinically applicable model with a restricted set of predictors where transparency and scalability is favored
- We will follow the Transparent reporting of a multivariable prediction model for individual prognosis or diagnosis (TRIPOD) guidelines[1] in the reporting of our study results
- The prediction models will be developed and validated in Swedish register data, and the extent to which they can be generalized is unclear: further work to validate the model in other national contexts will be necessary

For peer review only - http://bmjopen.bmj.com/site/about/guidelines.xhtml

INTRODUCTION

Suicide is among the leading causes of death among younger individuals.[2] Meanwhile, suicide is often accompanied, and/or driven, by underlying psychiatric disorders – mood disorders in particular.[2] Compared to the general population, depressed individuals have been found to have around a 20 times higher rate of suicide.[3] Antidepressants are the main pharmacological treatment option for mood disorders – taken by over 12% of the population in the US[4] – and SSRIs are the most common antidepressant type in many countries.[4 5] They are also the first-line pharmacological treatment for depression among children and adolescents in many countries, including in Sweden.[6]

Although commonly prescribed, there are concerns about the risk of suicide during SSRI treatment among children and adolescents, based on evidence from RCTs[7] and observational studies.[8] Questions on whether and in what circumstances this association indicates causation remain.[8] Regardless of causality, producing a model to assess which individuals are more or less likely to engage in suicidal behaviour after initiating SSRI medication could be an important aid in clinical decision-making, with models that predict suicidal behaviour risk within short time frames expected to be most clinically relevant.[9] However, applying a risk prediction model represents only one aspect of a more comprehensive approach necessary to prevent suicidal behaviour.[10]

Many studies have developed prediction models for suicide or suicidal behaviour in different populations.[11] However, no prior study has predicted suicidal behaviour among children, adolescents, and young adults who initiate an SSRI.

Some previous prediction studies have incorporated a wide variety of available predictors in order to harness the potential of large routinely collected electronic health records.[12-14] For example, a previous study using Swedish register data was able to predict suicide following a psychiatric inpatient visit with a relatively high level of discrimination performance.[13] However, this approach results in large models that may not be practical to implement in real-life settings where clinicians do not have access to routinely collected data. In such a setting, a clinician might want a restricted number of predictors to input into a risk prediction model that is simple, scalable, and transparently operationalized, for example as an online tool.[15 16]

Given the high baseline risk among many under consideration for SSRI-treatment, and the possibility of an elevated risk observed early during treatment,[9] we therefore aim to predict the risk of suicidal behaviour within 30 days of SSRI initiation in a population of children, adolescents, and young adults, aged 8-24 years. We do this in two ways: by applying a broad predictor set and relatively complex ensemble machine-learning approach, as compared to a clinically informed approach using a restricted set of predictors and favouring a more transparent model.

RESEARCH AIM

To predict the risk of suicidal behaviour among young individuals within 30 days of SSRI initiation, with a secondary analysis considering events within 90 days of initiation. We will use a two-stage approach in order to: A) explore the performance of an ensemble prediction model using machine learning methods

For peer review only - http://bmjopen.bmj.com/site/about/guidelines.xhtml

in Swedish register data, and to B) develop a clinically applicable model with a restricted set of predictors where transparency and scalability is favored.

METHODS AND ANALYSIS

We will use a two-stage approach to produce a prediction model:

A. *Broad predictor model*: a relatively broad set of predictors is included in a number of different machine learning base models, the predictions of which are then combined to produce an ensemble prediction.
B. *Restricted predictor model:* a restricted set of top predictors identified from model A is included in a number of different machine learning models, which are compared in terms of predictive performance and transparency.

Data source

We will obtain data from Swedish national registers, where linkage of records is based on unique personal identification numbers.[17] The Prescribed Drug Register – which includes all dispensed medications since July 2005 – will be used to extract information on psychotropic medication receipt, including the initiation of SSRI medication. [18] The Total Population Register, which includes demographic information on Swedish residents since 1968, will be used to extract sex and country of birth for an individual.[19] The National Patient Register, documenting discharge diagnoses from in- and outpatient care since 1973 and 2001 respectively, will be used for information on psychiatric diagnoses, including suicide attempts. [20] The Cause of Death Register will be used to extract information on death by suicide.[21] The Longitudinal Integrated Database for Health Insurance and Labour Market Studies (LISA) register – which includes information on socioeconomic variables for individuals aged 16 or above since 1990 and for those aged 15 or above since 2010 – will be used to extract information on the highest commenced education between individuals and their parents.[22] The multi-generation register, including information on family relations,[23] will be used to link individuals to their parents, whose history of psychiatric disease and of death by suicide can then be extracted from the National Patient Register and Cause of Death register, respectively.

Inclusion criteria

We will include all individuals aged 8-24 years who were born in Sweden and who initiated an SSRI after a wash-out period of 365 days free from any antidepressants during 1st January 2008 to 31st December 2020. The first initiation event per individual will be included.

Patient and public involvement

We have not involved patients in planning or executing this study. The research is based on observational register data, meaning no participant recruitment was necessary.

For peer review only - http://bmjopen.bmj.com/site/about/guidelines.xhtml

Outcome ascertainment

Based on previous work, we will consider any ICD-10 diagnosis code X60-X84 (suicidal behaviour of known intent) and Y10-Y34 (suicidal behaviour of unknown intent).[24 25] Only diagnoses made in specialist care are considered as our data linkage does not include primary care diagnoses.

We will consider suicide attempts or death by suicides (suicidal behaviour) within 30 days of SSRI initiation. As secondary analyses, we will a) consider only suicidal behaviour of known intent within 30 days, and b) consider suicidal behaviour within 90 days of SSRI initiation.

Predictors

Broad predictor model

We will include a broad range of predictors based on the availability in our register linkage.

The predictors include: sex; age at SSRI initiation; history of mental and behavioural disorder diagnoses, previous suicidal behaviour, and overdoses; psychotropic medications prescribed concurrently or within a year before initiation of the SSRI; highest commenced education between the index person and their parents in the year before SSRI initiation; prescriber practice type; parental history of mental and behavioural disorder diagnoses, suicidal behaviour, and overdoses (same codes as in individuals); and family history of death by suicide. See Table 1, and Table 2 for diagnosis definitions, and Table 3 for medication definitions.

All predictors aside from age will be considered as categorical variables. If there is evidence for it, we will consider accounting for non-linear effects of age in the LR model.

Restricted predictor model

We will include the following three predictors to ensure face-value validity of the prediction model: age, sex, and history of previous suicide attempt. We will identify the top 30 predictors that contributed most to predictive performance from the LR and XG base models used in the broad predictor model (model A). We will pool these predictors and include those deemed important based on clinical and statistical input, alongside the three face-value predictors. If there is evidence for it, we will consider accounting for non-linear effects of age in the LR model.

Missing values

We will exclude predictors where more than 20% of values are missing. In remaining predictors, if it is deemed that there is missingness at random (MAR), we will consider multiple imputation methods.[26]

Model validation and goodness of fit

Overall classification performance using area under the receiver operating characteristic curves (AUC) will be estimated for all models; 95% confidence intervals will be calculated using Delong's method.[13 27]

For peer review only - http://bmjopen.bmj.com/site/about/guidelines.xhtml

For the broad variable search model and for the selected restricted predictor model, we will assess the predictive performance of the trained models using: Concordance index, Brier score, and a calibration plot.[13 15]

For the broad variable search model and for the selected restricted predictor model, we will also asses sensitivity, specificity, positive and negative predictive values at the risk threshold of 1%, given that this is the approximate prevalence of suicidal behaviour within 30 days after SSRI initiation in Swedish registers.[25] That is, any child, adolescent, or young adult with a predicted risk greater than 1% have a risk that is higher than what is expected in the population, and can therefore be considered "high-risk".[15 28] We will also report calibration performance at a 5% risk cut-off.

For the final implementation of the model in clinical practice, we will provide a continuous risk score rather than a risk cut-off to provide a more flexible and individually-tailored aid to clinical decision-making.[28]

We will follow the transparent reporting of a multivariable prediction model for individual prognosis or diagnosis (TRIPOD) guidelines.[1]

Model development

Throughout the paper, we will employ different machine learning models: XGBoost (XG), neural net (NN), logistic regression (LR), and support vector machines (SVM).[13 29]

We will develop all models in a sample ("training sample") using all data from 1st January 2008- 31st December 2017.

We will validate all models in a sample ("testing sample") using all data from 1st January 2018-31st December 2020 (around 23% of the entire study period). That is, no random sampling was performed for either training or testing sample.

As a secondary validation, we will validate the final restricted model in the period 1st January 2018-28th February 2020 to see if the model performance was influenced by the COVID-19 pandemic.

Broad predictor model

For each model XG, NN, LR, and SVM, we will identify hyperparameters by a grid search with 10-fold cross validation. We will use the AUC as the evaluation criterion for hyperparameter tuning[29] – that is, we will choose the set of hyperparameters that maximize the AUC for each model.

After model tuning, we will combine the predictions from all the different models. The class probabilities of each model will be summed and averaged – the final prediction will consist of the class with the highest average probability. In effect, the predictions of each model will be weighted equally.

Finally, the 30 predictors that are most important (i.e. contributed most to the predictive performance) in each of the XG and LR base models will be extracted (predictors rankings are not available from the NN and SVM models).[30] The pooled list of these predictors will then be considered for inclusion in the restricted predictor model based on statistical and clinical input from the research group. In the case of

For peer review only - http://bmjopen.bmj.com/site/about/guidelines.xhtml

the LR model, predictive performance is judged by the absolute value of the beta coefficients on the log scale. In the case of the XG model, it was based on the predictor importance score.

Restricted predictor model

For each model XG, NN, LR, and SVM, we will identify hyperparameters by a grid search with 10-fold cross validation. We will use the AUC as the evaluation criterion for hyperparameter tuning.[29]

We will then compare the predictive performance of the different models when including the restricted set of predictors in the testing sample, using the Delong method to assess statistical significance of the difference. We will also produce an ensemble prediction in the same way as described for the broad predictor model. If there is no model that performs statistically significantly better than the others in terms of AUC score (including the ensemble model), we will consider the logistic regression model as the final restricted predictor model.[16] If there is a model with statistically significantly better AUC performance compared to the others (including the ensemble model), this model will be selected as the final restricted predictor model.

We favour the LR model in absence of clear superiority because this multivariate regression approach allows more transparency: the intercept and each predictor coefficient can be reported, allowing a prediction score for each study participant to be easily calculated by clinicians. This intercept of the model, which is a transformation of the baseline risk in the population, can also be changed to fit the particular population considered. In other words, an LR model would allow for greater transparency and scalability, including easy dissemination of the model as an online tool.[15]

In the final model, we will assess the prediction performance in terms of the AUC in the subset of individuals who received a diagnosis of depression (ICD-10 code F32 or F33) in the last 90 days.

Generalizability

Predictors will be defined in such a way that it will be possible to apply the models in other countries and find comparable predictors.

Ethics and dissemination

The research has been approved by the Swedish Ethical Review Authority (decision number 2020-06540). The use of anonymized register data waives the need to secure informed consent.[31] We will disseminate our findings by publishing in peer-reviewed open-access journals, and presenting at international conferences.

For peer review only - http://bmjopen.bmj.com/site/about/guidelines.xhtml

ACKNOWLEDGMENTS

Funding

This work was supported by the Swedish Research Council (grant number 2018-02213), and by the National Institute for Health and Care Research (NIHR) Applied Research Collaboration Oxford and Thames Valley at Oxford Health NHS Foundation Trust (grant number N/A). The views expressed are those of the authors and not necessarily those of the NHS, the NIHR or the Department of Health and Social Care. SV is funded by Forte (Swedish Research Council for Health, Working Life and Welfare, Postdoctoral Fellowship, grant number 2022-00824).

Author contributions

TL, ZC, and SF conceptualized the study. TL drafted the protocol. All authors provided critical input on protocol content and the protocol write-up. All authors fulfil authorship criteria as set out by International Committee of Medical Journal Editors (ICMJE).

Conflicts of interest statement

The authors report no conflicts of interest.

For peer review only

REFERENCES

1. Patzer RE, Kaji AH, Fong Y. TRIPOD reporting guidelines for diagnostic and prognostic studies. *JAMA surgery* 2021;156(7):675-76.
2. Fazel S, Runeson B. Suicide. *New England Journal of Medicine* 2020;382(3):266-74. doi: 10.1056/NEJMra1902944
3. Ösby U, Brandt L, Correia N, et al. Excess mortality in bipolar and unipolar disorder in Sweden. *Archives of general psychiatry* 2001;58(9):844-50.
4. Pratt LA, Brody DJ, Gu Q. Antidepressant Use among Persons Aged 12 and Over: United States, 2011-2014. NCHS Data Brief. Number 283. *National Center for Health Statistics* 2017
5. Abbing-Karahagopian V, Huerta C, Souverein PC, et al. Antidepressant prescribing in five European countries: application of common definitions to assess the prevalence, clinical observations, and methodological implications. *European journal of clinical pharmacology* 2014;70(7):849-57. doi: 10.1007/s00228-014-1676-z
6. Läkemedelsverket. Depression, ångestsyndrom och tvångssyndrom hos barn och vuxna - behandlingsrekommendation: Information från Läkemedelsverket. 2016;27(6):26–59.
7. Hammad TA, Laughren T, Racoosin J. Suicidality in Pediatric Patients Treated With Antidepressant Drugs. *Archives of general psychiatry* 2006;63(3):332-39.
8. Dragioti E, Solmi M, Favaro A, et al. Association of Antidepressant Use With Adverse Health Outcomes: A Systematic Umbrella Review. *JAMA Psychiatry* 2019;76(12):1241-55. doi: 10.1001/jamapsychiatry.2019.2859
9. Kirtley OJ, van Mens K, Hoogendoorn M, et al. Translating promise into practice: a review of machine learning in suicide research and prevention. *The Lancet Psychiatry* 2022;9(3):243-52.
10. Kessler RC, Bossarte RM, Luedtke A, et al. Suicide prediction models: a critical review of recent research with recommendations for the way forward. *Molecular psychiatry* 2019:1-12.
11. Belsher BE, Smolenski DJ, Pruitt LD, et al. Prediction models for suicide attempts and deaths: a systematic review and simulation. *JAMA psychiatry* 2019;76(6):642-51.
12. Gradus JL, Rosellini AJ, Horváth-Puhó E, et al. Prediction of sex-specific suicide risk using machine learning and single-payer health care registry data from Denmark. *JAMA psychiatry* 2020;77(1):25-34.
13. Chen Q, Zhang-James Y, Barnett EJ, et al. Predicting suicide attempt or suicide death following a visit to psychiatric specialty care: A machine learning study using Swedish national registry data. *PLoS medicine* 2020;17(11):e1003416.
14. Gradus JL, Rosellini AJ, Horváth-Puhó E, et al. Predicting sex-specific nonfatal suicide attempt risk using machine learning and data from Danish national registries. *American journal of epidemiology* 2021;190(12):2517-27.
15. Fazel S, Wolf A, Larsson H, et al. The prediction of suicide in severe mental illness: development and validation of a clinical prediction rule (OxMIS). *Translational psychiatry* 2019;9(1):1-10.
16. Whiting D, Fazel S. How accurate are suicide risk prediction models? Asking the right questions for clinical practice. *Evidence-based mental health* 2019;22(3):125-28.
17. Ludvigsson JF, Otterblad-Olausson P, Pettersson BU, et al. The Swedish personal identity number: possibilities and pitfalls in healthcare and medical research. *European journal of epidemiology* 2009;24(11):659-67.
18. Wettermark B, Hammar N, MichaelFored C, et al. The new Swedish Prescribed Drug Register—opportunities for pharmacoepidemiological research and experience from the first six months. *Pharmacoepidemiology and drug safety* 2007;16(7):726-35.

For peer review only - http://bmjopen.bmj.com/site/about/guidelines.xhtml

19. Ludvigsson JF, Almqvist C, Bonamy A-KE, et al. Registers of the Swedish total population and their use in medical research. *European journal of epidemiology* 2016;31(2):125-36.
20. Ludvigsson JF, Andersson E, Ekbom A, et al. External review and validation of the Swedish national inpatient register. *BMC public health* 2011;11(1):450.
21. Brooke HL, Talbäck M, Hörnblad J, et al. The Swedish cause of death register. *European journal of epidemiology* 2017;32(9):765-73.
22. Ludvigsson JF, Svedberg P, Olén O, et al. The longitudinal integrated database for health insurance and labour market studies (LISA) and its use in medical research. *European Journal of Epidemiology* 2019:1-15.
23. Ekbom A. The Swedish multi-generation register. Methods in Biobanking: Springer 2011:215-20.
24. Webb RT, Lichtenstein P, Larsson H, et al. Suicide, hospital-presenting suicide attempts, and criminality in bipolar disorder: examination of risk for multiple adverse outcomes. *The Journal of clinical psychiatry* 2014;75(8):e809.
25. Lagerberg T, Fazel S, Sjölander A, et al. Selective serotonin reuptake inhibitors and suicidal behaviour: a population-based cohort study. *Neuropsychopharmacology* 2021:1-7.
26. Horton NJ, Kleinman KP. Much ado about nothing: A comparison of missing data methods and software to fit incomplete data regression models. *The American Statistician* 2007;61(1):79-90.
27. DeLong ER, DeLong DM, Clarke-Pearson DL. Comparing the areas under two or more correlated receiver operating characteristic curves: a nonparametric approach. *Biometrics* 1988:837-45.
28. Wynants L, van Smeden M, McLernon DJ, et al. Three myths about risk thresholds for prediction models. *BMC medicine* 2019;17(1):1-7.
29. Tate AE, McCabe RC, Larsson H, et al. Predicting mental health problems in adolescence using machine learning techniques. *PloS one* 2020;15(4):e0230389.
30. Chen Q, Zhang-James Y, Barnett EJ, et al. Predicting suicide attempt or suicide death following a visit to psychiatric specialty care: A machine learning study using Swedish national registry data. *PLoS Medicine* 2020;17(11):1-19. doi: 10.1371/journal.pmed.1003416
31. Ludvigsson JF, Håberg SE, Knudsen GP, et al. Ethical aspects of registry-based research in the Nordic countries. *Clinical epidemiology* 2015;7:491.

For peer review only

For peer review only - http://bmjopen.bmj.com/site/about/guidelines.xhtml

TABLES

Table 1. Predictors included in the broad predictor model, by register	
**Total population register**	
Sex	
Native born	
**National patient register**	
Psychiatric diagnosis within 1 mo. before initiation	See Table 2 for diagnosis subdivisions.
Psychiatric diagnosis within 2 to 12 mo. before initiation	
Psychiatric diagnosis >12 mo. before initiation	
**Prescribed drug register**	
Age at initiation	1-year bands
Prescriber practice	Primary/Specialist/Psychiatric specialist
Psychiatric drug receipt within 3 mo. before initiation	See Table 3 for medication subdivisions.
Psychiatric drug receipt 3 mo.-12 mo. before initiation	
Psychiatric drug receipt >12 mo. before initiation	
**LISA**	
Highest commenced education of index individual's parents	Primary school, secondary school, university/vocational school
**Multi-generation register + national patient register**	
Family history of psychiatric disease among the index individual's parents: lifetime before initiation	See Table 2 for diagnosis subdivisions.
**Multi-generation register + cause of death register**	
Family history of death by suicide among index individual's parents: lifetime before initiation	All ICD-10 X60-X84; Y10-Y34.

For peer review only - http://bmjopen.bmj.com/site/about/guidelines.xhtml

Table 2. Diagnoses codes included for predictors	
**Diagnosis**	**ICD-10 code categorization**
**Mental and behavioural disorders**	
Organic mental disorder	F00-F09
Substance use disorder	F10-19
Schizophrenia and psychotic disorder	F20-F29
Manic episode	F30
Bipolar disorder	F31
Depressive episode	F32
Recurrent depressive episode	F33
Persistent mood disorder	F34
Other mood disorders	F38
Unspecified mood disorder	F39
Phobic anxiety disorder	F40.0-F40.2
Other anxiety disorders	F41.0-F41.1
OCD	F42
Reaction to severe stress, and adjustment disorders	F43
Hypochondriacal disorder	F452
Eating disorders	F50
Anorexia nervosa	F500, F501
Bulimia nervosa	F502, F503
Overeating / vomiting due to other psychological disorders	F504, F505
Other / unspecified eating disorders	F508, F509
Nonorganic sleep disorders	F51
Dissocial personality disorder	F602
Emotionally unstable personality disorder	F603
Pathological gambling	F630
Gender identity disorders	F64
Mental retardation	F70-F79
Autism	F840, F841, F845, F848, F849
Hyperkinetic disorder	F90
Conduct disorders	F91
Emotional disorders with onset specific to childhood	F93
Tic disorders	F95
**Suicidal behaviour**	X60-X84, Y10-Y34
**Overdose**	T36-51, X40-49

For peer review only - http://bmjopen.bmj.com/site/about/guidelines.xhtml

Table 3. ATC codes included for predictors

Medication	ATC code
Opioids and pain medication	N02A
Antiepileptic drugs	N03A
Antipsychotics	N05A
Anxiolytics, hypnotics, and sedatives	N05B excl. N05BA, N05C
Benzodiazepines	N05BA
Antidepressants, excluding SSRIs	N06A excl. N06AB
ADHD medication	N06B
Drugs used in addiction disorders	N07B

For peer review only

For peer review only - http://bmjopen.bmj.com/site/about/guidelines.xhtml

TRIPOD Checklist: Prediction Model Development and Validation

Section/Topic	Item		Checklist Item	Page
**Title and abstract**				
Title	1	D;V	Identify the study as developing and/or validating a multivariable prediction model, the target population, and the outcome to be predicted.	1
Abstract	2	D;V	Provide a summary of objectives, study design, setting, participants, sample size, predictors, outcome, statistical analysis, results, and conclusions.	2
**Introduction**				
Background and objectives	3a	D;V	Explain the medical context (including whether diagnostic or prognostic) and rationale for developing or validating the multivariable prediction model, including references to existing models.	3
	3b	D;V	Specify the objectives, including whether the study describes the development or validation of the model or both.	3
**Methods**				
Source of data	4a	D;V	Describe the study design or source of data (e.g., randomized trial, cohort, or registry data), separately for the development and validation data sets, if applicable.	4
	4b	D;V	Specify the key study dates, including start of accrual; end of accrual; and, if applicable, end of follow-up.	4
Participants	5a	D;V	Specify key elements of the study setting (e.g., primary care, secondary care, general population) including number and location of centres.	4
	5b	D;V	Describe eligibility criteria for participants.	4
	5c	D;V	Give details of treatments received, if relevant.	NA
Outcome	6a	D;V	Clearly define the outcome that is predicted by the prediction model, including how and when assessed.	5
	6b	D;V	Report any actions to blind assessment of the outcome to be predicted.	NA
Predictors	7a	D;V	Clearly define all predictors used in developing or validating the multivariable prediction model, including how and when they were measured.	5; Table 1-3
	7b	D;V	Report any actions to blind assessment of predictors for the outcome and other predictors.	NA
Sample size	8	D;V	Explain how the study size was arrived at.	4
Missing data	9	D;V	Describe how missing data were handled (e.g., complete-case analysis, single imputation, multiple imputation) with details of any imputation method.	5-7
Statistical analysis methods	10a	D	Describe how predictors were handled in the analyses.	5-7
	10b	D	Specify type of model, all model-building procedures (including any predictor selection), and method for internal validation.	5-7
	10c	V	For validation, describe how the predictions were calculated.	
	10d	D;V	Specify all measures used to assess model performance and, if relevant, to compare multiple models.	5-7
	10e	V	Describe any model updating (e.g., recalibration) arising from the validation, if done.	NA
Risk groups	11	D;V	Provide details on how risk groups were created, if done.	6
Development vs. validation	12	V	For validation, identify any differences from the development data in setting, eligibility criteria, outcome, and predictors.	NA
**Results**				
Participants	13a	D;V	Describe the flow of participants through the study, including the number of participants with and without the outcome and, if applicable, a summary of the follow-up time. A diagram may be helpful.	NA
	13b	D;V	Describe the characteristics of the participants (basic demographics, clinical features, available predictors), including the number of participants with missing data for predictors and outcome.	NA
	13c	V	For validation, show a comparison with the development data of the distribution of important variables (demographics, predictors and outcome).	NA
Model development	14a	D	Specify the number of participants and outcome events in each analysis.	NA
	14b	D	If done, report the unadjusted association between each candidate predictor and outcome.	NA
Model specification	15a	D	Present the full prediction model to allow predictions for individuals (i.e., all regression coefficients, and model intercept or baseline survival at a given time point).	NA
	15b	D	Explain how to the use the prediction model.	NA
Model performance	16	D;V	Report performance measures (with CIs) for the prediction model.	NA
Model-updating	17	V	If done, report the results from any model updating (i.e., model specification, model performance).	NA
**Discussion**				
Limitations	18	D;V	Discuss any limitations of the study (such as nonrepresentative sample, few events per predictor, missing data).	NA
Interpretation	19a	V	For validation, discuss the results with reference to performance in the development data, and any other validation data.	NA
	19b	D;V	Give an overall interpretation of the results, considering objectives, limitations, results from similar studies, and other relevant evidence.	NA
Implications	20	D;V	Discuss the potential clinical use of the model and implications for future research.	NA
**Other information**				
Supplementary information	21	D;V	Provide information about the availability of supplementary resources, such as study protocol, Web calculator, and data sets.	NA
Funding	22	D;V	Give the source of funding and the role of the funders for the present study.	8

*Items relevant only to the development of a prediction model are denoted by D, items relating solely to a validation of a prediction model are denoted by V, and items relating to both are denoted D;V. We recommend using the TRIPOD Checklist in conjunction with the TRIPOD Explanation and Elaboration document.

For peer review only - http://bmjopen.bmj.com/site/about/guidelines.xhtml

BMJ Open

Predicting risk of suicidal behaviour after initiation of selective serotonin reuptake inhibitors in children, adolescents, and young adults: protocol for development and validation of clinical prediction models

Journal:	*BMJ Open*
Manuscript ID	bmjopen-2023-072834.R1
Article Type:	Protocol
Date Submitted by the Author:	03-Jul-2023

Complete List of Authors:	Lagerberg, Tyra ; University of Oxford, Department of Psychiatry; Karolinska Institute, Department of Medical Epidemiology and Biostatistics<br>Virtanen, Suvi; Karolinska Institutet, Department of Medical Epidemiology and Biostatistics<br>Kuja-Halkola, Ralf; Karolinska Institutet, Department of Medical Epidemiology and Biostatistics<br>Hellner, Clara; Karolinska Institute, Clinical neuroscience<br>Lichtenstein, Paul; Karolinska Institutet, Department of Medical Epidemiology and Biostatistics<br>Fazel, Seena; University of Oxford, Department of Psychiatry; Oxford Health NHS Foundation Trust<br>Chang, Zheng; Karolinska Institutet, Department of Medical Epidemiology and Biostatistics

**Primary Subject Heading**:	Epidemiology
Secondary Subject Heading:	Mental health
Keywords:	Suicide & self-harm < PSYCHIATRY, Child & adolescent psychiatry < PSYCHIATRY, MENTAL HEALTH, REGISTRIES, Risk Factors

SCHOLARONE™ Manuscripts

Predicting risk of suicidal behaviour after initiation of selective serotonin reuptake inhibitors in children, adolescents, and young adults: protocol for development and validation of clinical prediction models

Tyra Lagerberg[1,2], Suvi Virtanen[1], Ralf Kuja-Halkola[1], Clara Hellner[3,4], Paul Lichtenstein[1], Seena Fazel[2,5], Zheng Chang[1]

[1] Department of Medical Epidemiology and Biostatistics, Karolinska Institutet, Stockholm, Sweden

[2] Department of Psychiatry, Warneford Hospital, University of Oxford, Oxford, United Kingdom

[3] Department of Clinical Neuroscience, Centre for Psychiatry Research, Karolinska Institutet

[4] Stockholm Health Care Services, Stockholm, Sweden

[5] Oxford Health NHS Foundation Trust

Corresponding author:

Tyra Lagerberg. Email: tyra.lagerberg@ki.se / tyra.lagerberg@psych.ox.ac.uk; Address: Department of Psychiatry, Warneford Hospital, University of Oxford, Oxford, United Kingdom OX3 7JX; Telephone: + 44 (0)1865 618200; ORCID: 0000-0003-2480-0216

Key words: prediction, selective serotonin reuptake inhibitors, suicidal behaviour, children and adolescents, young adults

Word count: 2,417

For peer review only - http://bmjopen.bmj.com/site/about/guidelines.xhtml

ABSTRACT

Introduction: There is concern regarding suicidal behaviour risk during selective serotonin reuptake inhibitor (SSRI) treatment among the young. A clinically useful model for predicting suicidal behaviour risk should have high predictive performance in terms of discrimination and calibration; transparency and ease of implementation are desirable.

Methods and analysis: Using Swedish national registers, we will identify individuals initiating an SSRI aged 8-24 years 2007-2020. We will develop: A) a model based on a broad set of predictors, and B) a model based on a restricted set of predictors. For the broad predictor model, we will consider an ensemble of four base models: XGBoost (XG), neural net (NN), elastic net logistic regression (EN), and support vector machine (SVM). The predictors with the greatest contribution to predictive performance in the base models will be determined. For the restricted predictor model, clinical input will be used to select predictors based on the top predictors in the broad model, and inputted in each of the XG, NN, EN, and SVM models. If any show superiority in predictive performance as defined by the area under the receiver-operator curve (AUC), this model will be selected as the final model; otherwise, the EN model will be selected. The training and testing samples will consist of data from 2007-2017 and 2018-2020, respectively. We will assess the final model performance in individuals receiving a depression diagnosis within 90 days before SSRI initiation.

The aims are to A) develop a model predicting suicidal behavior risk after SSRI initiation among children and youths, using machine learning methods, and B) develop a model with a restricted set of predictors, favoring transparency and scalability.

Ethics and dissemination: The research is approved by the Swedish Ethical Review Authority (2020-06540). We will disseminate findings by publishing in peer-reviewed open-access journals, and presenting at international conferences.

STRENGTHS AND LIMITATIONS OF THIS STUDY

- The study will make use of a large and nationally representative data linkage of Swedish registers, covering virtually any Swedish resident who initiated SSRI treatment during the study period
- We will compare different machine learning models and approaches to selecting predictors, in order to explore the performance of an ensemble prediction model using machine learning methods in Swedish register data, and to develop a clinically applicable model with a restricted set of predictors where transparency and scalability is favored
- We will follow the Transparent reporting of a multivariable prediction model for individual prognosis or diagnosis (TRIPOD) guidelines in the reporting of our study results
- The prediction models will be developed and validated in Swedish register data, and the extent to which they can be generalized is unclear: further work to validate the model in other national contexts will be necessary

For peer review only - http://bmjopen.bmj.com/site/about/guidelines.xhtml

INTRODUCTION

Suicide is among the leading causes of death among younger individuals.[1] Meanwhile, suicide is often accompanied, and/or driven, by underlying psychiatric disorders – mood disorders in particular.[1] Compared to the general population, depressed individuals have been found to have around a 20 times higher rate of suicide.[2] Antidepressants are the main pharmacological treatment option for mood disorders – taken by over 12% of the population in the US [3] – and SSRIs are the most common antidepressant type in many countries.[3, 4] They are also the first-line pharmacological treatment for depression among children and adolescents in many countries, including in Sweden.[5]

Although commonly prescribed, there are concerns about the risk of suicide during SSRI treatment among children and adolescents, based on evidence from RCTs [6] and observational studies.[7] Questions on whether and in what circumstances this association indicates causation remain.[7] Regardless of causality, producing a model to assess which individuals, out of those initiating an SSRI, are more or less likely to engage in suicidal behaviour could be an important aid in clinical decision-making in terms of determining which individuals will require more clinical oversight. Models that predict suicidal behaviour risk within short time frames are expected to be most clinically relevant.[8] However, applying a risk prediction model represents only one aspect of a more comprehensive approach necessary to prevent suicidal behaviour.[9]

Many studies have developed prediction models for suicide or suicidal behaviour in different populations.[10] However, no prior study has predicted suicidal behaviour among children, adolescents, and young adults who initiate an SSRI.

Some previous prediction studies have incorporated a wide variety of available predictors in order to harness the potential of large routinely collected electronic health records.[11-13] For example, a previous study using Swedish register data was able to predict suicide following a psychiatric inpatient visit with a relatively high level of discrimination performance.[12] However, this approach results in large models that may not be practical to implement in real-life settings where clinicians do not have access to routinely collected data. In such a setting, a clinician might want a restricted number of predictors to input into a risk prediction model that is simple, scalable, and transparently operationalized, for example as an online tool.[14, 15] Previous research also suggests that clinicians want to understand why specific individuals are assigned a specific risk score, in particular when the risk classification is counterintuitive,[16] which may favour the latter type of model.

Given the high baseline risk among many under consideration for SSRI-treatment, and the possibility of an elevated risk observed early during treatment,[8] we therefore aim to predict the risk of suicidal behaviour within 30 days of SSRI initiation in a population of children, adolescents, and young adults, aged 8-24 years. We do this in two ways: by applying a broad predictor set and relatively complex ensemble machine-learning approach, as compared to a clinically informed approach using a restricted set of predictors and favouring a more transparent model. It should be noted that our aim is not to determine which individuals should or should not be treated with an SSRI, but to identify whom, among those who do initiate an SSRI, is at particularly elevated risk of suicidal behaviour. Given that children under age 18 are routinely treated for psychiatric disorders in specialist care in Sweden, we expect our model to be mainly applicable to a specialist care setting.

For peer review only - http://bmjopen.bmj.com/site/about/guidelines.xhtml

RESEARCH AIM

To predict the risk of suicidal behaviour among young individuals within 30 days of SSRI initiation, with a secondary analysis considering events within 90 days of initiation. We will use a two-stage approach in order to: A) explore the performance of an ensemble prediction model using machine learning methods in Swedish register data, and to B) develop a clinically applicable model with a restricted set of predictors where transparency and scalability is favored.

METHODS AND ANALYSIS

We will use a two-stage approach to produce a prediction model:

A. *Broad predictor model*: a relatively broad set of predictors is included in a number of different machine learning base models, the predictions of which are then combined to produce an ensemble prediction.
B. *Restricted predictor model:* a restricted set of top predictors identified from model A is included in a number of different machine learning models, which are compared in terms of predictive performance and transparency.

Data source

We will obtain data from Swedish national registers, where linkage of records is based on unique personal identification numbers.[17] The Prescribed Drug Register – which includes all dispensed medications since July 2005 – will be used to extract information on psychotropic medication receipt, including the initiation of SSRI medication.[18] The Total Population Register, which includes demographic information on Swedish residents since 1968, will be used to extract sex and country of birth for an individual.[19] The National Patient Register, documenting discharge diagnoses from in- and outpatient care since 1973 and 2001 respectively, will be used for information on psychiatric diagnoses, including suicide attempts.[20] The Cause of Death Register will be used to extract information on death by suicide.[21] The Longitudinal Integrated Database for Health Insurance and Labour Market Studies (LISA) register – which includes information on socioeconomic variables for individuals aged 16 or above since 1990 and for those aged 15 or above since 2010 – will be used to extract information on the highest commenced education between individuals and their parents.[22] The multi-generation register, including information on family relations,[23] will be used to link individuals to their parents, whose history of psychiatric disease and of death by suicide can then be extracted from the National Patient Register and Cause of Death register, respectively.

Inclusion criteria

We will include all individuals aged 8-24 years who were born in Sweden and who initiated an SSRI after a wash-out period of 365 days free from any antidepressants during 1st January 2007 to 31st December 2020. The first initiation event per individual will be included.

For peer review only - http://bmjopen.bmj.com/site/about/guidelines.xhtml

Patient and public involvement

We have not involved patients in planning or executing this study. The research is based on observational register data, meaning no participant recruitment was necessary.

Outcome ascertainment

Based on previous work, we will consider any ICD-10 diagnosis code X60-X84 (suicidal behaviour of known intent) and Y10-Y34 (suicidal behaviour of unknown intent).[24, 25] Only diagnoses made in specialist care are considered as our data linkage does not include primary care diagnoses.

We will consider suicide attempts or death by suicides (suicidal behaviour) within 30 days of SSRI initiation. As secondary analyses, we will a) consider only suicidal behaviour of known intent within 30 days, and b) consider suicidal behaviour within 90 days of SSRI initiation.

Predictors

Broad predictor model

We will include a broad range of predictors based on the availability in our register linkage.

The predictors include: sex; age at SSRI initiation; history of mental and behavioural disorder diagnoses, previous suicidal behaviour, and overdoses; psychotropic medications; highest commenced education between the index person and their parents in the year before SSRI initiation; prescriber practice type; parental history of mental and behavioural disorder diagnoses, suicidal behaviour, and overdoses (same codes as in individuals); and family history of death by suicide. See Table 1, and Table 2 for diagnosis definitions, and Table 3 for medication definitions.

All predictors aside from age will be considered as categorical variables. If there is evidence for it, we will consider accounting for non-linear effects of age in the EN model.

Restricted predictor model

We will include the following three predictors to ensure face-value validity of the prediction model: age, sex, and history of previous suicide attempt. We will identify the top 30 predictors that contributed most to predictive performance in each of the base models in stage A (see section Model Development). We will pool these predictors and include those deemed important based on clinical input, alongside the three face-value predictors.

Missing values

We will exclude predictors where more than 20% of values are missing. In remaining predictors, if it is deemed that there is missingness at random (MAR), we will consider multiple imputation methods.[26]

For peer review only - http://bmjopen.bmj.com/site/about/guidelines.xhtml

Model validation and goodness of fit

Overall classification performance using area under the receiver operating characteristic curves (AUC) will be estimated for all models; 95% confidence intervals will be calculated using Delong's method.[12, 27]

For the broad variable search model and for the selected restricted predictor model, we will assess the predictive performance of the trained models using: Concordance index, Brier score, and a calibration plot.[12, 14]

For the broad variable search model and for the selected restricted predictor model, we will also asses sensitivity, specificity, positive and negative predictive values at the risk threshold of 1%, given that this is the approximate prevalence of suicidal behaviour within 30 days after SSRI initiation in Swedish registers.[25] That is, any child, adolescent, or young adult with a predicted risk greater than 1% have a risk that is higher than what is expected in the population, and can therefore be considered "high-risk".[14, 28] We will also report calibration performance at a 2% and 5% risk cut-off.

The impact of different risk score cut-offs could be further evaluated in future work – however, continuous risk scores could provide a more flexible idea of individual risk as compared to risk cut-offs.[28]

We will follow the transparent reporting of a multivariable prediction model for individual prognosis or diagnosis (TRIPOD) guidelines.[29]

Model development

Throughout the paper, we will employ different machine learning models: XGBoost (XG), neural net (NN), elastic net logistic regression (EN), and support vector machines (SVM).[12, 30]

We will develop all models in a sample ("training sample") using all data from 1st January 2007- 31st December 2017. We will apply shrinkage procedures where appropriate, for example by employing an elastic net logistic regression.

We will validate all models in a sample ("testing sample") using all data from 1st January 2018-31st December 2020. That is, no random sampling was performed for either training or testing sample.

As a secondary validation, we will validate the final restricted model in the period 1st January 2018-28th February 2020 to see if the model performance was influenced by the COVID-19 pandemic.

Broad predictor model

For each model XG, NN, EN, and SVM, we will identify hyperparameters by a grid search with 10-fold stratified cross-validation, ensuring an approximately equal proportion of individuals with the outcome in each fold. We will use the AUC as the evaluation criterion for hyperparameter tuning [30] – that is, we will choose the set of hyperparameters that maximize the AUC for each model.

For peer review only - http://bmjopen.bmj.com/site/about/guidelines.xhtml

After model tuning, we will combine the predictions from all the different models. The class probabilities of each model will be summed and averaged – the final prediction will consist of the class with the highest average probability (that is, we will produce an ensemble prediction). In effect, the predictions of each model will be weighted equally.

Finally, the 30 predictors that are most important (i.e. contributed most to the predictive performance) in each of the base models (when applied in the training data) will be extracted using permutation based feature importance, if computationally feasible. The pooled list of these predictors will then be considered for inclusion in the restricted predictor model based on clinical input and the available evidence base on the risk factors; the original set of pooled predictors will be presented, and we will describe our reasoning for the final predictor inclusion in the paper.

If permutation-based feature importance is not computationally feasible, we will consider the most important predictors based on the predictor importance score from the XG model, and absolute beta coefficients from the EN model.

Restricted predictor model

For each model XG, NN, EN, and SVM, we will identify hyperparameters by a grid search with 10-fold stratified cross-validation, ensuring an approximately equal proportion of individuals with the outcome in each fold. We will use the AUC as the evaluation criterion for hyperparameter tuning.[30]

We will then compare the predictive performance of the different models when including the restricted set of predictors in the testing sample, using the Delong method to assess statistical significance of the difference. We will also produce an ensemble prediction in the same way as described for the broad predictor model. If there is no model that performs better than the others in terms of AUC score (including the ensemble model) and calibration performance, we will consider the elastic net logistic regression model as the final restricted predictor model.[15] If there is a model with statistically significantly better AUC performance compared to the others (including the ensemble model), this model will be selected as the final restricted predictor model.

We favour the EN model in absence of clear superiority because this multivariate regression approach allows more transparency: the intercept and each predictor coefficient can be reported, allowing a prediction score for each study participant to be easily calculated by clinicians. This intercept of the model, which is a transformation of the baseline risk in the population, can also be changed to fit the particular population considered. In other words, an EN model would allow for greater transparency and scalability, including easy dissemination of the model as an online tool.[14]

Given the parameter selection inherent in an EN model, it is possible that even in the broad predictor model step it produces a model with fewer than 30 predictors. If this model already at the stage of the broad predictor model has a predictive performance superior to or equal to the other models, we will consider bypassing stage B of this protocol and using this model as the final model.

In the final model, we will assess the prediction performance in terms of the AUC in the subset of individuals who received a diagnosis of depression (ICD-10 code F32 or F33) in the last 90 days. In the group of individuals aged 18-24 years, we will also consider testing the model performance separately for individuals initiating their SSRI in primary and specialist care settings.

For peer review only - http://bmjopen.bmj.com/site/about/guidelines.xhtml

Generalizability

Predictors have been defined in such a way that it will be possible to apply the models in other countries and find comparable predictors, for example by using standard ICD-10 and ATC codes to define diagnosis and medication predictors, respectively. While our time-based validation allows some generalizability over time in the Swedish context, external validation from other national settings could be a next step.

Ethics and dissemination

The research has been approved by the Swedish Ethical Review Authority (decision number 2020-06540). The use of anonymized register data waives the need to secure informed consent.[31] We will disseminate our findings by publishing in peer-reviewed open-access journals, and presenting at international conferences.

ACKNOWLEDGMENTS

Funding

This work was supported by the Swedish Research Council (grant number 2018-02213), and by the National Institute for Health and Care Research (NIHR) Applied Research Collaboration Oxford and Thames Valley at Oxford Health NHS Foundation Trust (grant number N/A). The views expressed are those of the authors and not necessarily those of the NHS, the NIHR or the Department of Health and Social Care. SV is funded by Forte (Swedish Research Council for Health, Working Life and Welfare, Postdoctoral Fellowship, grant number 2022-00824).

Author contributions

TL, ZC, and SF conceptualized the study. TL drafted the protocol. All authors provided critical input on protocol content and the protocol write-up. All authors fulfil authorship criteria as set out by International Committee of Medical Journal Editors (ICMJE).

Conflicts of interest statement

The authors report no conflicts of interest.

For peer review only - http://bmjopen.bmj.com/site/about/guidelines.xhtml

REFERENCES

1. Fazel, S. and B. Runeson, *Suicide.* New England Journal of Medicine, 2020. **382**(3): p. 266-274.
2. Ösby, U., et al., *Excess mortality in bipolar and unipolar disorder in Sweden.* Archives of general psychiatry, 2001. **58**(9): p. 844-850.
3. Pratt, L.A., D.J. Brody, and Q. Gu, *Antidepressant Use among Persons Aged 12 and Over: United States, 2011-2014. NCHS Data Brief. Number 283.* National Center for Health Statistics, 2017.
4. Abbing-Karahagopian, V., et al., *Antidepressant prescribing in five European countries: application of common definitions to assess the prevalence, clinical observations, and methodological implications.* Eur J Clin Pharmacol, 2014. **70**(7): p. 849-57.
5. Läkemedelsverket, *Depression, ångestsyndrom och tvångssyndrom hos barn och vuxna - behandlingsrekommendation: Information från Läkemedelsverket.* 2016. **27**(6): p. 26–59.
6. Hammad, T.A., T. Laughren, and J. Racoosin, *Suicidality in Pediatric Patients Treated With Antidepressant Drugs.* Archives of general psychiatry, 2006. **63**(3): p. 332-339.
7. Dragioti, E., et al., *Association of Antidepressant Use With Adverse Health Outcomes: A Systematic Umbrella Review.* JAMA Psychiatry, 2019. **76**(12): p. 1241-1255.
8. Kirtley, O.J., et al., *Translating promise into practice: a review of machine learning in suicide research and prevention.* The Lancet Psychiatry, 2022. **9**(3): p. 243-252.
9. Kessler, R.C., et al., *Suicide prediction models: a critical review of recent research with recommendations for the way forward.* Molecular psychiatry, 2019: p. 1-12.
10. Belsher, B.E., et al., *Prediction models for suicide attempts and deaths: a systematic review and simulation.* JAMA psychiatry, 2019. **76**(6): p. 642-651.
11. Gradus, J.L., et al., *Prediction of sex-specific suicide risk using machine learning and single-payer health care registry data from Denmark.* JAMA psychiatry, 2020. **77**(1): p. 25-34.
12. Chen, Q., et al., *Predicting suicide attempt or suicide death following a visit to psychiatric specialty care: A machine learning study using Swedish national registry data.* PLoS medicine, 2020. **17**(11): p. e1003416.
13. Gradus, J.L., et al., *Predicting sex-specific nonfatal suicide attempt risk using machine learning and data from Danish national registries.* American journal of epidemiology, 2021. **190**(12): p. 2517-2527.
14. Fazel, S., et al., *The prediction of suicide in severe mental illness: development and validation of a clinical prediction rule (OxMIS).* Translational psychiatry, 2019. **9**(1): p. 1-10.
15. Whiting, D. and S. Fazel, *How accurate are suicide risk prediction models? Asking the right questions for clinical practice.* Evidence-based mental health, 2019. **22**(3): p. 125-128.
16. Yarborough, B.J.H., et al., *Clinical implementation of suicide risk prediction models in healthcare: a qualitative study.* BMC psychiatry, 2022. **22**(1): p. 789.
17. Ludvigsson, J.F., et al., *The Swedish personal identity number: possibilities and pitfalls in healthcare and medical research.* European journal of epidemiology, 2009. **24**(11): p. 659-667.
18. Wettermark, B., et al., *The new Swedish Prescribed Drug Register—opportunities for pharmacoepidemiological research and experience from the first six months.* Pharmacoepidemiology and drug safety, 2007. **16**(7): p. 726-735.

For peer review only - http://bmjopen.bmj.com/site/about/guidelines.xhtml

19. Ludvigsson, J.F., et al., *Registers of the Swedish total population and their use in medical research.* European journal of epidemiology, 2016. **31**(2): p. 125-136.
20. Ludvigsson, J.F., et al., *External review and validation of the Swedish national inpatient register.* BMC public health, 2011. **11**(1): p. 450.
21. Brooke, H.L., et al., *The Swedish cause of death register.* European journal of epidemiology, 2017. **32**(9): p. 765-773.
22. Ludvigsson, J.F., et al., *The longitudinal integrated database for health insurance and labour market studies (LISA) and its use in medical research.* European Journal of Epidemiology, 2019: p. 1-15.
23. Ekbom, A., *The Swedish multi-generation register*, in *Methods in Biobanking*. 2011, Springer. p. 215-220.
24. Webb, R.T., et al., *Suicide, hospital-presenting suicide attempts, and criminality in bipolar disorder: examination of risk for multiple adverse outcomes.* The Journal of clinical psychiatry, 2014. **75**(8): p. e809.
25. Lagerberg, T., et al., *Selective serotonin reuptake inhibitors and suicidal behaviour: a population-based cohort study.* Neuropsychopharmacology, 2021: p. 1-7.
26. Horton, N.J. and K.P. Kleinman, *Much ado about nothing: A comparison of missing data methods and software to fit incomplete data regression models.* The American Statistician, 2007. **61**(1): p. 79-90.
27. DeLong, E.R., D.M. DeLong, and D.L. Clarke-Pearson, *Comparing the areas under two or more correlated receiver operating characteristic curves: a nonparametric approach.* Biometrics, 1988: p. 837-845.
28. Wynants, L., et al., *Three myths about risk thresholds for prediction models.* BMC medicine, 2019. **17**(1): p. 1-7.
29. Patzer, R.E., A.H. Kaji, and Y. Fong, *TRIPOD reporting guidelines for diagnostic and prognostic studies.* JAMA surgery, 2021. **156**(7): p. 675-676.
30. Tate, A.E., et al., *Predicting mental health problems in adolescence using machine learning techniques.* PloS one, 2020. **15**(4): p. e0230389.
31. Ludvigsson, J.F., et al., *Ethical aspects of registry-based research in the Nordic countries.* Clinical epidemiology, 2015. **7**: p. 491.

For peer review only

For peer review only - http://bmjopen.bmj.com/site/about/guidelines.xhtml

TABLES

Table 1. Predictors included in the broad predictor model, by register	
**Total population register**	
Sex	
Native born	
**National patient register**	
Psychiatric diagnosis within 3 mo. before initiation	See Table 2 for diagnosis subdivisions.
Psychiatric diagnosis >3 mo. before initiation	
**Prescribed drug register**	
Age at initiation	1-year bands
Prescriber practice	Primary/Specialist/Psychiatric specialist
Psychiatric drug receipt within 3 mo. before initiation	See Table 3 for medication subdivisions.
Psychiatric drug receipt >3 mo. before initiation	
**LISA**	
Highest commenced education of index individual's parents	Primary school, secondary school, university/vocational school
**Multi-generation register + national patient register**	
Family history of psychiatric disease among the index individual's parents: lifetime before initiation	See Table 2 for diagnosis subdivisions.
**Multi-generation register + cause of death register**	
Family history of death by suicide among index individual's parents: lifetime before initiation	All ICD-10 X60-X84; Y10-Y34.

For peer review only - http://bmjopen.bmj.com/site/about/guidelines.xhtml

Table 2. Diagnoses codes included for predictors	
**Diagnosis**	**ICD-10 code categorization**
**Mental and behavioural disorders**	
Organic mental disorder	F00-F09
Substance use disorder	F10-19
Schizophrenia and psychotic disorder	F20-F29
Manic episode	F30
Bipolar disorder	F31
Depression	F32, F33
Persistent mood disorder	F34
Other or unspecified mood disorders	F38, F39
Phobic anxiety disorder	F40.0-F40.2
Other anxiety disorders	F41.0-F41.1
OCD	F42
Reaction to severe stress, and adjustment disorders	F43
Anorexia nervosa	F500, F501
Bulimia nervosa	F502, F503
Nonorganic sleep disorders	F51
Dissocial personality disorder or emotionally unstable personality disorder	F602, F603
Gender identity disorders	F64
Mental retardation	F70-F79
Autism	F840, F841, F845, F848, F849
Hyperkinetic disorder	F90
Conduct disorders	F91
**Suicidal behaviour**	X60-X84, Y10-Y34
**Overdose**	T36-51, X40-49

For peer review only

For peer review only - http://bmjopen.bmj.com/site/about/guidelines.xhtml

Table 3. ATC codes included for predictors

Medication	ATC code
Opioids and pain medication	N02A
Antiepileptic drugs	N03A
Antipsychotics	N05A
Anxiolytics, hypnotics, and sedatives	N05B excl. N05BA, N05C
Benzodiazepines	N05BA
Antidepressants, excluding SSRIs	N06A excl. N06AB
ADHD medication	N06B
Drugs used in addiction disorders	N07B

For peer review only

For peer review only - http://bmjopen.bmj.com/site/about/guidelines.xhtml

TRIPOD Checklist: Prediction Model Development and Validation

Section/Topic	Item		Checklist Item	Page
**Title and abstract**				
Title	1	D;V	Identify the study as developing and/or validating a multivariable prediction model, the target population, and the outcome to be predicted.	1
Abstract	2	D;V	Provide a summary of objectives, study design, setting, participants, sample size, predictors, outcome, statistical analysis, results, and conclusions.	2
**Introduction**				
Background and objectives	3a	D;V	Explain the medical context (including whether diagnostic or prognostic) and rationale for developing or validating the multivariable prediction model, including references to existing models.	3
	3b	D;V	Specify the objectives, including whether the study describes the development or validation of the model or both.	3, 4
**Methods**				
Source of data	4a	D;V	Describe the study design or source of data (e.g., randomized trial, cohort, or registry data), separately for the development and validation data sets, if applicable.	4
	4b	D;V	Specify the key study dates, including start of accrual; end of accrual; and, if applicable, end of follow-up.	5
Participants	5a	D;V	Specify key elements of the study setting (e.g., primary care, secondary care, general population) including number and location of centres.	4,5
	5b	D;V	Describe eligibility criteria for participants.	5
	5c	D;V	Give details of treatments received, if relevant.	NA
Outcome	6a	D;V	Clearly define the outcome that is predicted by the prediction model, including how and when assessed.	5
	6b	D;V	Report any actions to blind assessment of the outcome to be predicted.	NA
Predictors	7a	D;V	Clearly define all predictors used in developing or validating the multivariable prediction model, including how and when they were measured.	5; Table 1-3
	7b	D;V	Report any actions to blind assessment of predictors for the outcome and other predictors.	NA
Sample size	8	D;V	Explain how the study size was arrived at.	4,5
Missing data	9	D;V	Describe how missing data were handled (e.g., complete-case analysis, single imputation, multiple imputation) with details of any imputation method.	6
Statistical analysis methods	10a	D	Describe how predictors were handled in the analyses.	5-7
	10b	D	Specify type of model, all model-building procedures (including any predictor selection), and method for internal validation.	5-7
	10c	V	For validation, describe how the predictions were calculated.	
	10d	D;V	Specify all measures used to assess model performance and, if relevant, to compare multiple models.	5-7
	10e	V	Describe any model updating (e.g., recalibration) arising from the validation, if done.	NA
Risk groups	11	D;V	Provide details on how risk groups were created, if done.	6
Development vs. validation	12	V	For validation, identify any differences from the development data in setting, eligibility criteria, outcome, and predictors.	NA
**Results**				
Participants	13a	D;V	Describe the flow of participants through the study, including the number of participants with and without the outcome and, if applicable, a summary of the follow-up time. A diagram may be helpful.	NA
	13b	D;V	Describe the characteristics of the participants (basic demographics, clinical features, available predictors), including the number of participants with missing data for predictors and outcome.	NA
	13c	V	For validation, show a comparison with the development data of the distribution of important variables (demographics, predictors and outcome).	NA
Model development	14a	D	Specify the number of participants and outcome events in each analysis.	NA
	14b	D	If done, report the unadjusted association between each candidate predictor and outcome.	NA
Model specification	15a	D	Present the full prediction model to allow predictions for individuals (i.e., all regression coefficients, and model intercept or baseline survival at a given time point).	NA
	15b	D	Explain how to the use the prediction model.	NA
Model performance	16	D;V	Report performance measures (with CIs) for the prediction model.	NA
Model-updating	17	V	If done, report the results from any model updating (i.e., model specification, model performance).	NA
**Discussion**				
Limitations	18	D;V	Discuss any limitations of the study (such as nonrepresentative sample, few events per predictor, missing data).	NA
Interpretation	19a	V	For validation, discuss the results with reference to performance in the development data, and any other validation data.	NA
	19b	D;V	Give an overall interpretation of the results, considering objectives, limitations, results from similar studies, and other relevant evidence.	NA
Implications	20	D;V	Discuss the potential clinical use of the model and implications for future research.	NA
**Other information**				
Supplementary information	21	D;V	Provide information about the availability of supplementary resources, such as study protocol, Web calculator, and data sets.	NA
Funding	22	D;V	Give the source of funding and the role of the funders for the present study.	8

*Items relevant only to the development of a prediction model are denoted by D, items relating solely to a validation of a prediction model are denoted by V, and items relating to both are denoted D;V. We recommend using the TRIPOD Checklist in conjunction with the TRIPOD Explanation and Elaboration document.

For peer review only
